# The role of adipose tissue in liver fat accumulation: a sex-specific analysis in an exploratory cross-sectional study

**DOI:** 10.1186/s12944-025-02809-x

**Published:** 2025-12-01

**Authors:** Jana Hoffmann, Jochen Schmidt, Jens Thiele, Stefan Kwast, Roberto Falz, Timm Denecke, Martin Busse, Hans-Jonas Meyer

**Affiliations:** 1 Department of Diagnostic and Interventional Radiology, University of Leipzig, Leipzig, 04103 Germany; 2 Department of Neurophysics, Max Planck Institute for Human Cognitive and Neurosciences, Leipzig, 04103 Germany; 3 Department of Radiology, Helios Klinik Schkeuditz, Schkeuditz, 04435 Germany; 4 Helios Health Institute, Berlin, 13125 Germany; 5 Human-Machine-Interaction, Magdeburg-Stendal University of Applied Sciences, Magdeburg, 39114 Germany; 6 Outpatient Clinic of Sports Medicine, Leipzig, 04109 Germany

**Keywords:** MASLD, VAT, Subcutaneous fat, Obesity

## Abstract

**Introduction:**

Visceral (VAT), subcutaneous (SFT), and total body fat (FM) contribute to hepatic steatosis, yet their relative and sex-specific effects across total, regional, and site-specific levels remain unclear. We investigated associations between fat depots, standardized skinfold sites, and liver fat (LF) while adjusting for key metabolic covariates.

**Methods:**

In this secondary data analysis of a cross-sectional study, 48 adults (50% women; 49.6 ± 20.9 y; BMI 25.7 ± 3.7 kg/m²) underwent quantitative MRI to assess VAT and LF and ultrasound-based body mapping to quantify total and regional SFT as well as standardized skinfold sites. Bioelectrical impedance analysis determined FM. Regression analyses were conducted with LF as the dependent variable, identifying and controlling for significant covariates (diabetes mellitus [DM], arterial hypertension [aHT], hypercholesterolemia [HC], physical activity, age) (partial r). The Lindeman–Merenda–Gold (LMG) method decomposed total R² into fat-specific contributions. Total fat depots were adjusted for body surface area (BSA).

**Results:**

All regression models examining associations with LF showed total r values ranging from 0.63 to 0.79. In men, DM was the only significant covariate (p = 0.002). Values are given as: partial r, LMG share (% of R^2^). LF correlated with VAT/BSA (0.40, 51%), total SFT/BSA (0.38, 42%), and FM/BSA (0.36, 51%). Regionally, upper-body SFT (0.40, 45%) and SFT_arms (0.37, 47%) contributed most, whereas lower-body SFT (0.35, 13%) showed minimal impact. The triceps skinfold was the most influential site among skinfolds (0.45, 50%). In women, HC was the only significant covariate (p = 0.02). LF correlated with VAT/BSA (0.40, 49%), total SFT/BSA (0.51, 27%), and FM/BSA (0.47, 36%). Regional models yielded upper-body SFT (0.43, 36%), SFT_arms (0.38, 23%), and lower-body SFT (0.41, 9%). Among single sites, the umbilical skinfold was most relevant (0.43, 36%), followed by the biceps (0.42, 33%).

**Conclusion:**

VAT remains pivotal for LF in both sexes, yet SFT exhibits clear sex- and region-specific relevance. Only subcutaneous fat of the upper-body and arms contributed meaningfully to liver fat. Simple skinfold assessments—particularly triceps in men—may serve as practical indicator for early risk stratification. Larger, prospective cohorts are needed to confirm these findings.

**Trial registration:**

Not applicable this study did not involve any health care intervention.

**Supplementary Information:**

The online version contains supplementary material available at 10.1186/s12944-025-02809-x.

## Introduction

 Hepatic fat accumulation is increasingly recognized as both a marker and mediator of cardiometabolic risk, not only in individuals with overweight or obesity, but also in metabolically at-risk individuals with normal body weight [1]. Metabolic dysfunction-associated steatotic liver disease (MASLD), recently redefined to better capture its pathophysiologic basis, now affects up to 38% of the global population [2]. It often progresses silently toward steatohepatitis, fibrosis, or cirrhosis and is closely associated with cardiometabolic profiles such as type 2 diabetes mellitus, and dyslipidemia [3]. Despite its rising prevalence, MASLD remains widely undertreated—partly due to an incomplete understanding of determinants of hepatic fat accumulation. Several genetic polymorphisms contribute to inter-individual variation in liver fat content; however, they explain only a modest proportion of the total variance [4].

Among modifiable, extrahepatic factors, total body fat and its distribution have emerged as a key driver of disease progression [5, 6]. In particular, visceral adipose tissue (VAT) has been implicated in hepatic steatosis due to its direct drainage into the portal circulation, which delivers free fatty acids and pro-inflammatory mediators to the liver [7]. By contrast, subcutaneous adipose tissue (SAT) is often considered metabolically benign or even protective [8], but its role is increasingly recognized as context-dependent, as inflammation, fibrosis, or hormonal changes—particularly in postmenopausal women—can lead to redistribution of lipids toward visceral compartments [7, 9]. Sex-based differences in fat storage and hepatic lipid handling are well established [10]. Women tend to store more fat subcutaneously and exhibit more efficient hepatic fatty acid oxidation, whereas men accumulate more VAT and re-esterify free fatty acids more readily [11]. These physiological and hormonal distinctions emphasize the importance of sex-stratified analyses to clarify how different fat depots contribute to hepatic lipid accumulation.

According to clinical guidelines (e.g., European Association for the Study of the Liver (EASL), American Association for the Study of Liver Diseases (AASLD)), population-wide screening for hepatic steatosis is not currently recommended [12]. However, sex-specific risk stratification within at-risk groups—such as individuals with diabetes or obesity—is increasingly being discussed [13].

Despite growing evidence linking adipose depots to hepatic steatosis, several knowledge gaps remain: First, most prior studies rely on single-slice surrogates rather than total-volume measurement of VAT, potentially obscuring depot-specific effects [14]. Second, recent work suggests that subcutaneous fat may also exhibit region-dependent behavior, with upper- and lower-body adiposity showing distinct relationships to liver fat [15]. To address this complexity, SFT should be assessed at multiple hierarchical levels—whole-body, regional (upper- and lower-body), and localized skinfold sites—to provide a comprehensive overview of fat–liver interactions alongside VAT and total body fat. Third, sex-stratified analyses remain scarce, limiting our understanding of how adipose distribution differentially influences hepatic fat accumulation [10]. Lastly, data on early or subclinical stages of steatosis are limited. It remains unclear to what extent hepatic fat accumulation in participants with mild obesity may already be influenced by depot-specific fat volumes. While metabolic comorbidities are strongly associated with liver fat content, their role as effect modifiers should be also considered in fat depot analysis [16].

The present study addresses these gaps by examining sex-specific associations between liver fat and body fat depots in adults with diverse metabolic profiles ranging from normal weight to class I obesity. In a structured approach, we aimed to: (i) assess total body fat (FM), VAT, and total subcutaneous fat (SFT) and, (ii) to further delineate regional SFT components (upper- and lower-body) as well as localized subcutaneous fat layers at standardized skinfold sites to investigate their association with liver fat.

## Participants and Methods

### Study design

This is a secondary data analysis of a previous cross-sectional study aimed to examine the influence of FM, VAT, SFT on liver fat accumulation in both male and female participants with and without cardiometabolic diseases.

All participants were informed about the study’s objectives and procedures via a comprehensive information letter. Written informed consent was obtained from all individual participants prior to participation. The study was approved by the Ethics Committee of the Medical Faculty of the University of Leipzig (Approval IDs: 097/17-ek and 089/18-ek) and conducted and reported in accordance with the Declaration of Helsinki and STROBE guidelines [17].

As part of the study protocol, a medical history was obtained, documenting diagnosed diseases, current medications, habitual physical activity, and daily routines. Anthropometric measurements, including height and weight, were taken. A hierarchical assessment framework was implemented to characterize adiposity across multiple analytical levels, ranging from whole-body to site-specific measurements. First, total FM was quantified using bioelectrical impedance analysis (BIA) to represent overall adiposity, serving as the macroscopic phenotype from which individual fat depots originate. Second, the two principal fat compartments contributing to FM—VAT and SFT— were analyzed separately, acknowledging their distinct anatomical localization and metabolic relevance in hepatic lipid metabolism. VAT was quantified via magnetic resonance imaging (MRI), whereas SFT was assessed using a detailed, ultrasound-based body mapping protocol [18]. Third, in recognition of the structural and functional heterogeneity of SFT, this compartment was further subdivided into regional depots (upper- and lower-body) to investigate region-specific associations with liver fat. Finally, standardized skinfold sites established by the International Society for the Advancement of Kinanthropometry (ISAK: biceps, triceps, umbilical, femoral) were examined to quantify localized subcutaneous fat layers with direct clinical applicability.

This study represents a secondary analysis of data obtained within the framework of a previous study protocol [18]. The original study aimed to characterize abdominal composition and included standardized balanced fast field echo (bFFE)-based MRI acquisitions. For the present analysis, these data were complemented by an additional mDixonQuant subset that was not included in the main analysis. This was conducted solely to confirm agreement between liver fat quantification methods (bFFE vs. mDixonQuant), minimizing potential systematic bias to ensure robustness and methodological comparability. The BMI and hepatic fat ranges of this validation subsample were comparable to those observed in the main study cohort, supporting its representativeness.

#### *Sample* size

Through the utilization of G-Power software (Version 3.1.9.2, available at https://g-power.apponic.com/), a priori sample size calculation was performed using the following parameters: linear bivariate regression: one group, size of slope, α < 0.05. To achieve a statistical power of 0.8, and assuming a slope of 0.003 LF(%)/VAT (ml) with *r* = 0.53 [19], a sample size of 23 participants per group (male and female) was computed.

#### Participants

The cohort comprised 48 adults (mean age 49.6 ± 20.9 years), with a balanced split of age (52% aged ≤ 50 years, 48% >50 years). We included adults (>18 years) with and without metabolic diseases and a BMI ranging from 18.5 up to 34.9 kg/m^2^ (class 1 obesity) for analyses of hepatic fat content. The BMI distribution was as follows: 46% participants with normal weight (18.5–24.9 kg/m²), 39% with overweight (25.0–29.9 kg/m²), and 15% with class I obesity (30.0–34.9 kg/m²). This population is particularly relevant, as individuals with early-stage obesity, especially those with metabolic comorbidities, are at increased risk for developing hepatic steatosis [13].

Among male participants (*n* = 24), diabetes mellitus (DM), arterial hypertension (aHT), and hypercholesterolemia (HC) were present in 9, 12, and 6 participants, respectively; among female participants (*n* = 24), the corresponding counts were 5, 10, and 6. DM and aHT were based on documented diagnoses or current use of antidiabetic or antihypertensive medications, respectively. HC was defined by either a reported diagnosis of HC or current treatment with lipid-lowering agents (e.g., statins).

Participants concurrently enrolled in other interventional studies or with active infectious diseases such as hepatitis, liver cirrhosis, liver fibrosis, siderosis, hemochromatosis or edema, recent pregnancies, metallic implants, or cardiac devices were excluded. The descriptive data are presented in Table 1.

**Table 1:** Descriptive overview of the investigated cohort

	*Total cohort*	*Men*	*Women*	*Ranges*	p-value*
	*mean ± SD*	*mean ± SD*	*mean ± SD*		
	[*n*= 48]	[*n*= 24]	[*n*= 24]		
*Age (y)*	49.6 ± 20.9	50.18 ± 22.2	49.2 ± 20.68	22.0–78.0	0.888
*Height (cm)*	170.9 ± 9.8	177.4 ± 7.87	164.56 ± 7.4	149.0–188.0	< 0.0001
*Weight (kg)*	74.9 ± 11.5	80.8 ± 6.79	69.1 ± 10.6	56.0–106.0	< 0.0001
*BMI (kg/m* ^*2*^ *)*	25.7 ± 3.7	25.7 ± 3.2	25.76 ± 4.3	18.5–33.8	0.924
*18.5–24.9*	22 (46%)	10 (42%)	12 (50%)	-	-
*25.0–29.9.0.9*	19 (39%)	12 (50%)	7 (29%)	-	-
*30.0–34.9.0.9*	7 (15%)	2 (8%)	5 (21%)	-	-
*FM (%)*	27.3 ± 9.2	21.9 ± 6.7	32.76 ± 8.4	12.6–42.6	< 0.0001
*Physical Activity (exercise sessions/week)*	1.9 ± 1.1	2.1 ± 0.9	1.7 ± 1.3	0–4	0.086
*DM*	14 (29%)	9 (37%)	5 (21%)	-	0.341
*aHT*	22 (45%)	12 (50%)	10 (42%)	-	0.772
*HC*	12 (25%)	6 (25%)	6 (25%)	-	0.999

Data are presented as mean values

BMI categories did not differ significantly between men and women (Chi² test, *p* = 0.25)

*FM* total body fat, *SD* standard deviation, *BMI* body mass index,* DM* diabetes mellitus, *aHT* arterial hypertension,* HC * hypercholesterolemia

* t-test with α<0.05 and chi-square test for diagnoses between sexes

#### Bioelectrical impedance analysis (BIA)

In this study, FM was assessed using Body Comp Software 8.5 Professional (MEDICAL HealthCare GmbH, www.medi-cal.de) with a single-frequency segmental BIA 101 Anniversary Sport Edition (Akern srl, Florence, Italy). Electrodes were placed on each hand and foot while the participant was in a supine position. This setup allows for a comprehensive body composition assessment due to the distinct electrical properties of various tissue types. A low-level electrical current (800 µA) was passed through the body, and the resistance encountered was used to estimate FM through a proprietary algorithm [18]. 

#### MRI

All acquisitions were performed using a Philips Achieva 1.5T clinical MRI scanner (Philips Health care, Hamburg, Germany). Imaging was performed using a whole-body coil (dStream Torso coil) with a gradient strength of 33mT/m and a maximum gradient slew rate of 122T/m/s, covering the region from the diaphragm to the symphysis. Participants were positioned in a supine position.

#### Visceral fat assessment

A cross-sectional two-dimensional single-shot turbo spin-echo sequence (SSH-TSE) was used for VAT measurement. Single-shot sampling of k-space data was achieved by choosing a sufficiently short repetition time (TR = 818 ms), allowing for a short scan time (12 s) and minimizing the occurrence of flow and ghosting artifacts. The echo time (TE) was set to 80ms. Measurements were carried out using breath-hold gating. The acquisition was performed separately for the lower and upper abdomen to ensure complete coverage of both areas. 60 slices were used to quantify total visceral fat. The MRI protocol has been described in detail in a previous study [20].

For image evaluation, the PACS JiveX software (Visus Health IT GmbH, Bochum Germany; www.visus.com) was applied. For precision purposes, we manually delineated the intra-abdominal cavity and the bowel structures. The VAT area was then calculated by subtracting the bowel area from the total intra-abdominal area. To obtain the VAT mass, the resulting area was multiplied by the slice thickness (10 mm) and the assumed fat density [21]. This way, the calculated volume is deduced from a signal average across the slice profile. We assume the bias from non-uniform slice profiles and partial volume effects across slices to be negligible considering the extent of the regions of interest and the large slice thickness used.

#### Liver fat assessment

The MRI protocol from a previous framework [18] to quantify VAT included an additional gradient echo sequence bFFE (also known as TrueFISP or FIESTA), a standard for abdominal imaging. It offers high signal-to-noise ratios, strong tissue contrast, and reduced motion artifacts, advantageous for liver imaging in clinical settings [22]. Due to availability and low scan times, it is often preferred and has been demonstrated to be particularly useful in liver imaging and enable valid fat and water separation [23]. Liver fat estimates were obtained from this sequence via signal ratio analysis, incorporating bias correction specifically optimized for the constraints inherent to the acquisition protocol (flip angle = 20° [24], TR = 120 ms, 10 echo times). A detailed description of MRI protocol and bias correction procedure can be found in the supplementary material. Given the physical properties, we used a combined approach in this secondary data analysis: (i) documenting methodological and physical implementation (see supplementary material), and (ii) applying a comparative rule-out procedure in a subsample using a Dixon-based reference (mDixonQuant) to identify potential systematic biases.

**Fig. 1:**
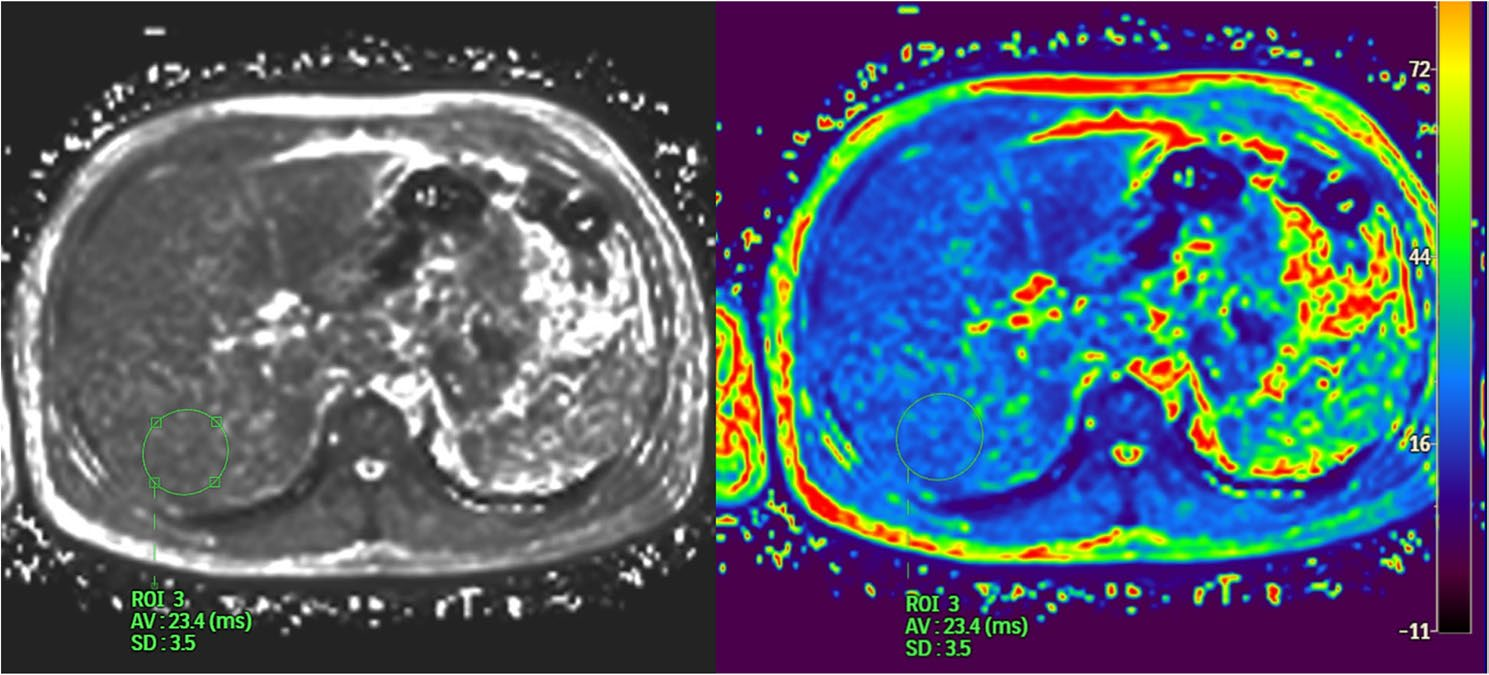
Liver fat quantification via mDixonQuant. ROI placement in the right lobe of the liver-preferably segment VII. The color scale visualizes T2^*^ of tissues, derived from their fat and water signal components

This comparative analysis between bias-corrected liver fat values derived from the signal model and reference measurements obtained using the mDixonQuant sequence (TR ≈ 6.5 ms, ΔTE ≈ 0.8 ms, flip angle = 5°, Philips MRI systems) included a cohort of 14 participants (Fig. 1). LF values were classified into grades S0–S3 according to Qadri et al. [25]. Additionally, intra-observer reliability of MRI-based liver fat quantification was assessed.

#### Subcutaneous fat tissue (SFT)

In this study, we used SFT to denote total and regional subcutaneous fat, rather than SAT, which is more commonly associated with abdominal subcutaneous adipose tissue. We applied a systematic, ultrasound-based body mapping protocol as described in a previous work [18]. Ultrasound was deliberately chosen for SFT assessment, as it enables high-resolution quantification of subcutaneous fat layers at peripheral and regionally delineated sites that are less practical to assess comprehensively by MRI. This approach allowed us to evaluate SFT at multiple hierarchical levels—whole-body, regional (upper- and lower-body fat), and standardized skinfold sites (in cm) defined by ISAK (biceps, triceps, umbilical, femoral)—providing a structured overview of how subcutaneous fat relates to liver fat accumulation beyond a single-compartment perspective. Upper-body referred to the trunk region (excluding arms), arms were investigated separately, and lower-body comprised the leg and gluteal regions.

Moreover, this approach reflects a clinically oriented design, aiming to characterize fat distribution with methods that are practical, accessible, and potentially applicable for early risk assessment in routine clinical care.

### Statistical analysis

All analyses were performed in SPSS (v23; IBM Corp., Armonk, NY, USA) and Python (v3.10.0; Python Software Foundation). Descriptive data are shown as mean ± SD. Data and imaging results were screened for plausibility, with outliers identified via the ROUT method. Sex differences were tested using independent t-tests for continuous variables and chi-square tests for categorical variables. Agreement between hepatic fat quantification methods was assessed with the intraclass correlation coefficient (ICC, two-way mixed model, absolute agreement), and Bland–Altman analysis (mean bias ± SD and 95% limits of agreement). ICC values < 0.50 were interpreted as poor, 0.50–0.75 as moderate, 0.75–0.90 as good, and >0.90 as excellent agreement [26]. Methodological equivalence was assumed when the mean bias was within ± 1.5%, in line with previous research [27, 28].

All analyses were stratified by sex. Associations between LF and total adipose compartments were examined in separate linear regression models (one-sided, α = 0.05), with fat depots normalized to body surface area (BSA). As we a priori expected all fat depots to be positively associated with liver fat (LF), one-tailed significance tests were applied. The strength of association was expressed as correlation coefficients (r) and coefficients of determination (R²). Confounder-adjusted associations are reported as partial correlation coefficients (partial r). Covariates (DM, aHT, HC, age, and physical activity) were evaluated for inclusion using forward regression to identify relevant confounders and to avoid model overfitting. Variables that were not statistically significant but considered biologically relevant for liver fat associations were retained if their inclusion changed the total r of the model by ≥ 0.10 [29]. For transparency, models including all covariates are presented in the supplementary material. The Lindeman–Merenda–Gold (LMG) method decomposed total R² into independent shares to estimate each predictor’s contribution (fat depot and covariate) [30]. No statistical correction for multiple testing was applied, as analyses were performed separately for each fat depot and were not based on conditional or repeated hypothesis testing.

## Results

### Reliability and agreement between LF quantification methods

In a validation subset of 14 participants, liver fat estimates derived from bias-corrected bFFE and mDixonQuant showed excellent agreement (ICC = 0.94; Pearson *r* = 0.98, *p* < 0.001) and a mean bias of − 0.91 ± 0.88% (3.71 ± 3.59% vs. 4.62 ± 3.81%), with 95% limits of agreement ranging from − 2.64 to + 0.81%. The relative difference (1.22-fold), with mDixonQuant yielding higher values than bFFE, closely mirrored the 1.24-fold discrepancy reported between MR spectroscopy or biopsy-derived values and mDixon fat estimates in prior studies [28]. This difference falls well within the expected inter-method variability across imaging modalities (typically ± 1.5%) [27, 28]. Additionally, bFFE measurements showed excellent intrarater reliability (ICC = 0.97).

### Characteristics of sex-specific fat compartments

Descriptive body composition data are shown in Table 2. One male participant with grade-3 steatosis (≈ 30% LF) was identified during screening; this case is reported in the steatosis table but excluded from correlation and variance analyses to prevent leverage effects. Another male participant was excluded due to localized hepatic fat inhomogeneity. Women had significantly higher FM and SFT than men (both *p* < 0.0001), while VAT did not differ between sexes, resulting in a higher VAT/SFT ratio in men.

**Table 2:** Absolute and relative values of body fat parameters

	*Total cohort*	*Men*	*Women*	*Ranges*	p-value*
	[*n*= 46]	[*n*= 22]	[*n*= 24]		
Total Fat Depots					
VAT [kg]	3.24 ± 2.43	3.53 ± 2.68	2.96 ± 2.20	0.35–10.04	0.429
VAT [%]	4.17 ± 2.96	4.32 ± 3.13	4.02 ± 2.85	0.49–15.66	0.736
VAT/BSA [kg/m^2^]	1.72 ± 1.25	1.79 ± 1.34	1.65 ± 1.21	0.17–3.95	0.430
SFT [kg]	17.16 ± 7.12	12.98 ± 4.23	21.00 ± 7.12	6.33–34.60	< 0.0001
SFT [%]	23.40 ± 9.00	16.13 ± 4.45	29.78 ± 6.72	8.06–45.18	< 0.0001
SFT/BSA [kg/m^2^]	9.35 ± 3.89	6.60 ± 2.08	11.87 ± 3.53	3.09–20.01	< 0.0001
VAT/SFT	0.19 ± 0.15	0.25 ± 0.17	0.13 ± 0.09	0.02–0.73	0.006
FM [kg]	20.23 ± 8.13	16.76 ± 6.16	23.24 ± 8.71	10.90–46.80	0.007
FM [%]	27.15 ± 9.47	21.15 ± 6.44	32.66 ± 8.45	12.60–46.80	< 0.0001
FM/BSA [kg/m^2^]	10.95 ± 4.25	8.66 ± 3.00	13.04 ± 4.18	4.35–19.92	0.0002
LF [%]	4.99 ± 4.36	4.42 ± 3.33	5.52 ± 5.14	1.43–16.04	0.390
Steatosis grading †					
*S0 (< 5.75%)*	34 (72%)	17 (74%)	17 (71%)	-	-
*S1 (≥ 5.75%)*	10 (21%)	5 (22%)	5 (21%)	-	-
*S2 (≥ 15.50%)*	2 (4%)	0	2 (8%)	-	-
*S3 (≥ 21.35%)‡*	1 (2%)	1 (4%)	0	-	-
Regional Fat Depots (kg)					
SFT_arms	2.00 ± 0.95	1.59 ± 0.64	2.38 ± 1.06	0.72–3.90	0.0036
SFT_upperBody	6.98 ± 3.82	5.65 ± 3.12	8.19 ± 4.13	1.38–17.15	0.0227
SFT_lowerBody	8.18 ± 3.50	5.74 ± 1.31	10.43 ± 3.47	3.92–21.09	< 0.0001
Skinfold Sites (cm)					
SFT_biceps	0.82 ± 0.53	0.56 ± 0.36	1.07 ± 0.55	0.17–2.00	0.0007
SFT_triceps	1.15 ± 0.58	0.78 ± 0.40	1.49 ± 0.53	0.34–3.01	< 0.0001
SFT_umbilical	2.12 ± 1.20	1.59 ± 0.97	2.61 ± 1.22	0.48–3.93	0.0029
SFT_femoral	1.19 ± 0.57	0.80 ± 0.29	1.55 ± 0.53	0.29–2.51	< 0.0001

Values are presented as mean and standard deviation; FM, fat mass in %, LF, liver fat; VAT, visceral adipose tissue (% is related to body weight); SFT, subcutaneous fat tissue; SFT_biceps, biceps skinfold fat depth; SFT_triceps, triceps skinfold fat depth; SFT_umbilical, umbilical skinfold fat depth; SFT_femoral, femoral skinfold fat depth; BSA: body surface area, VAT/SFT: ratio of visceral adipose tissue and total subcutaneous fat mass

*unpaired t-test between men and women with α < 0.05, S0-S3, steatosis grades; † Qadri et al. criteria, ‡ This single male S3 case (LF ≈ 30%) is displayed for completeness but is excluded from all correlation and variance analyses

**Fig. 2:**
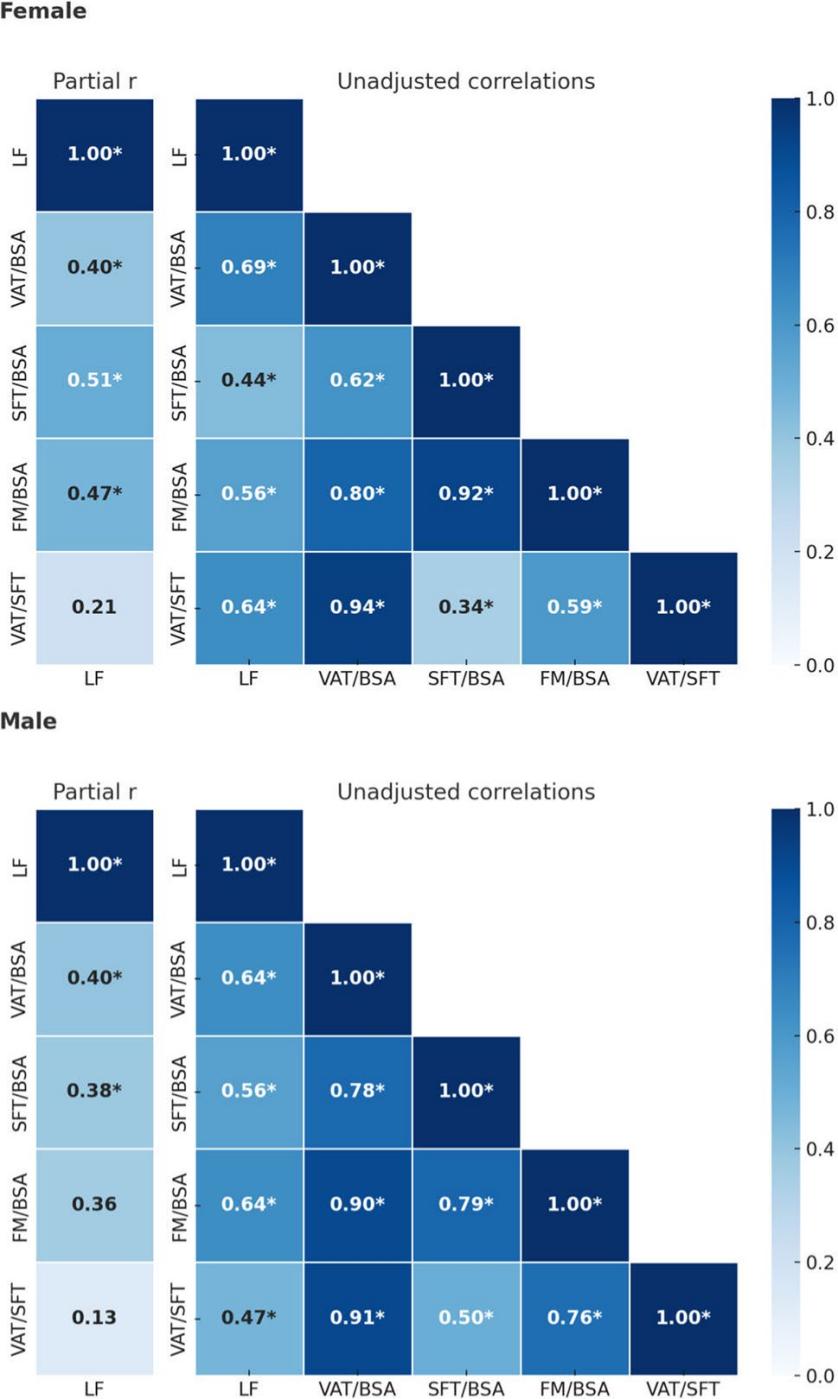
Bias-adjusted and unadjusted correlation matrices of body composition components. The upper panel (female) shows correlations adjusted for hypercholesterolemia (HC) and the corresponding unadjusted values; the lower panel (male) shows correlations adjusted for diabetes mellitus (DM) and their unadjusted counterparts. Numbers and the color intensity represent the strength of Pearson’s correlation coefficient. * significant deviation from zero with *p*<0.05, one-sided

Figure2 shows the correlation matrix, comparing unadjusted and confounder-adjusted associations between hepatic fat and adipose depots. Table 3 presents total R² and the proportion contributed by covariates. Age did not improve model fit but is included in Table 3 for transparency in evaluating sex-specific patterns. 

**Table 3:** Sex-specific Pearson correlations and contributions (LMG) of adipose tissue depots and metabolic factors to liver fat

Model	Total	Partial	Total	Portion of Total *R*² (LMG)		
	*r*	*r*	*R*²	Fat depot	Covariate	Age
Male (*n* = 22)					DM	
*Total Fat Depots*						
VAT/BSA + DM	0.70*	0.40*	0.49	0.25 (51%)	0.24 (49%)	-
VAT/BSA + DM + Age	0.77*	0.54*	0.59	0.27 (46%)	0.20 (33%)	0.12 (21%)
SFT/BSA + DM	0.70*	0.38*	0.49	0.20 (42%)	0.28 (58%)	-
SFT/BSA + DM + Age	0.70*	0.35	0.49	0.17 (34%)	0.26 (52%)	0.07 (14%)
FM/BSA + DM	0.69*	0.36	0.47	0.24 (51%)	0.23 (49%)	-
FM/BSA + DM + Age	0.69*	0.31	0.48	0.20 (43%)	0.20 (43%)	0.07 (14%)
VAT/SFT + DM	0.64*	0.13	0.41	0.12 (28%)	0.29 (70%)	-
VAT/SFT + DM + Age	0.65*	−0.03	0.42	0.08 (19%)	0.27 (64%)	0.07 (17%)
*Regional Fat Depots (kg)*						
SFT_arms + DM	0.69*	0.37	0.47	0.22 (47%)	0.25 (53%)	-
SFT_upperBody + DM	0.70*	0.40*	0.49	0.22 (45%)	0.27 (55%)	-
SFT_lowerBody + DM	0.69*	0.35	0.47	0.06 (13%)	0.41 (87%)	-
*Skinfold Sites (cm)*						
SFT_biceps + DM	0.63*	0.06	0.40	0.08 (20%)	0.32 (80%)	-
SFT_triceps + DM	0.72*	0.45*	0.52	0.26 (50%)	0.26 (50%)	-
SFT_umbilical + DM	0.67*	0.31	0.46	0.14 (30%)	0.32 (70%)	-
SFT_femoral + DM	0.64*	0.11	0.40	0.02 (5%)	0.38 (95%)	-
				VAT	SFT	DM
VAT + SFT_upperBody + SFT_arms + DM	0.72*	-	0.52	0.16 (31%)	0.18 (35%)	0.18 (34%)
VAT/BSA + SFT_triceps + DM	0.74*	-	0.55	0.19 (34%)	0.15 (27%)	0.21 (38%)
Female (*n* = 24)					HC	
*Total Fat Depots*						
VAT/BSA + HC	0.75*	0.40*	0.57	0.28 (49%)	0.29 (51%)	-
VAT/BSA + HC + Age	0.75*	0.25	0.56	0.21 (37%)	0.24 (43%)	0.11 (19%)
SFT/BSA + HC	0.79*	0.51*	0.62	0.17 (27%)	0.46 (73%)	-
SFT/BSA + HC + Age	0.79*	0.44*	0.63	0.12 (19%)	0.38 (61%)	0.13 (20%)
FM/BSA + HC	0.77*	0.47*	0.60	0.21 (36%)	0.39 (64%)	-
FM/BSA + HC + Age	0.78*	0.38*	0.60	0.15 (25%)	0.34 (56%)	0.11 (19%)
VAT/SFT + HC	0.71*	0.21	0.51	0.21 (42%)	0.29 (58%)	-
VAT/SFT + HC + Age	0.73*	0.05	0.54	0.16 (30%)	0.26 (48%)	0.12 (22%)
*Regional Fat Depots (kg)*						
SFT_arms + HC	0.75*	0.38*	0.56	0.13 (23%)	0.43 (77%)	-
SFT_upperBody + HC	0.76*	0.43*	0.58	0.21 (36%)	0.37 (64%)	-
SFT_lowerBody + HC	0.76*	0.41*	0.57	0.05 (9%)	0.52 (91%)	-
*Skinfold Sites (cm)*						
SFT_biceps + HC	0.76*	0.42*	0.58	0.19 (33%)	0.39 (67%)	-
SFT_triceps + HC	0.70*	0.13	0.49	0.01 (2%)	0.48 (98%)	-
SFT_umbilical + HC	0.76*	0.43*	0.58	0.21 (36%)	0.37 (64%)	-
SFT_femoral + HC	0.73*	0.30	0.53	0.03 (6%)	0.5 (94%)	-
				VAT	SFT	HC
VAT + SFT_upperBody + SFT_arms + HC	0.77*	-	0.60	0.20 (33%)	0.14 (23%)	0.26 (44%)
VAT/BSA + SFT_umbilical + HC	0.76*	-	0.59	0.19 (33%)	0.14 (23%)	0.25 (43%)

Total r: correlation of the combination of depot plus confounder; Total R²: LF variance explained by the combination of depot plus confounder; partial r: confounder- adjusted correlation; portion of Total R²: LF variance explained by this specific variable calculated via LMG method; SFT_biceps, subcutaneous fat depth at biceps skinfold site; SFT_triceps, subcutaneous fat depth at triceps skinfold site; SFT_umbilical, subcutaneous fat depth at umbilical skinfold site; SFT_femoral, subcutaneous fat depth at femoral skinfold site

*VAT* visceral adipose tissue, *SFT* total subcutaneous fat, *VAT/SFT* visceral-to-subcutaneous fat ratio, *FM* whole-body fat mass, *DM* diabetes mellitus, *HC* hypercholesterolemia

* significant values, α < 0.05, one-sided

### General predictive values of covariates

In the extended analysis, neither physical activity, age, nor arterial hypertension improved the overall model fit substantially (Δr < 0.1). Similar findings were observed for diabetes mellitus in women and hypercholesterolemia in men. The complete combined model including all covariates is provided in the supplementary material.

#### (i) Total fat depots vs. LF

In men, DM was the sole significant covariate in LF associations (*p* = 0.002), while in women HC was the main significant covariate (*p* = 0.02). In both sexes, LF correlated positively with adiposity measures, especially visceral and subcutaneous fat. In men, associations of VAT/BSA and SFT/BSA with LF remained significant but weaker after adjusting for DM, while FM/BSA lost significance (*p* = 0.06). The VAT/SFT ratio was not independently associated with LF despite a significant unadjusted link. In women, LF was significantly associated with all fat depots; after adjustment for HC, all remained moderately correlated, with SFT/BSA showing the strongest association (*r* = 0.51). The VAT/SFT ratio showed only a weak, non-significant relationship. In men, VAT/BSA and DM each explaining about half the variance. A similar pattern was seen for SFT/BSA, though DM contributed slightly more. Among women, the total SFT depot showed limited unique variance in liver fat, with HC providing the predominant contribution (27% vs. ~ 73%). In the VAT model, contributions were more balanced. Age showed no significant value in either sex.

#### (ii) Regional fat depots and skinfold sites vs. LF

In men, upper-body SFT showed the strongest association with liver fat, while arm SFT exhibited a comparable magnitude. Both, upper-body and arm SFT accounted for roughly half of the explained variance. In contrast, lower-body SFT displayed clearly weaker associations and only a minimal independent contribution to liver fat. Among single sites, the triceps skinfold was the only clearly influential anthropometric marker, outperforming the umbilical site and markedly exceeding biceps and femoral measurements. Both the biceps and femoral sites showed weak associations, and the femoral site contributed negligibly.

In women, upper-body SFT emerged as the most relevant regional depot, with SFT of the arms providing relevant, though smaller, contributions, while lower-body SFT exhibited minimal unique variance. For single sites, the umbilical— and, to a lesser extent, the biceps— skinfolds were the only measures that showed meaningful associations with liver fat, whereas triceps was comparatively uninformative and the femoral site remained minor.

## Discussion

This study provides a comprehensive, sex-stratified evaluation of adipose tissue compartments in relation to hepatic fat accumulation in adults. Three key findings emerged: (1) VAT was a central determinant of LF in both sexes; in our models, VAT and the primary metabolic covariate each accounted for roughly half of the explained variance in LF. (2) SFT showed a sex-specific role: in men, total SFT contributed a similar share to LF variance as VAT and diabetes mellitus, challenging the VAT-centric view. In women, SFT correlated with LF but contributed a smaller unique share, with hypercholesterolemia predominating. Importantly, SFT effects were region dependent, with upper-body fat and arm depots being most relevant to liver fat accumulation, particularly in men, whereas lower-body SFT had limited independent relevance in both sexes. Consistent with this regional pattern, single skinfolds mirrored regional depot differences. (3) The key metabolic comorbidity significantly influencing liver fat differed by sex: diabetes mellitus in men and hypercholesterolemia in women exerted influential contributions.

### Overall fat mass and liver fat

Regarding the confounder-adjusted FM–LF association, we found a moderate relationship in both sexes. FM, as a composite measure of VAT and SFT, did not result in a stronger partial correlation with LF than either evaluated separately in both sexes.

In women, both the correlation and explained variance for FM closely mirrored the correlation mean values of VAT and SFT, which is also reflected in their overall contribution to MAFLD risk. In men, the FM–LF association was slightly weaker than that of its individual components; however, its overall contribution was greater, reflecting the additional role of SFT. Nevertheless, the findings are consistent with previous population-based research reporting that FM is associated with higher LF in both sexes [31]. Similarly, Hatamizargaran et al. demonstrated that higher body fat percentages, even in individuals with normal weight, were linked to increased non-alcoholic fatty liver disease (NAFLD) incidence in both sexes [32]. Conversely, a large-scale study of Lin et al. found that in adults over 65 years, metabolic factors, not FM, were more strongly associated with NAFLD, whereas in younger individuals, BMI and FM showed stronger associations [33]. Although age itself was not a significant confounder in our analyses, the relevance of metabolic comorbidities in our cohort may be consistent with the findings of Lin et al., as metabolic risk factors generally become more prevalent with advancing age.

### Visceral fat and liver fat – A consistent link in both sexes

Our results confirm that, even at relatively early stages of hepatic steatosis in adults with obesity, greater visceral adiposity is associated with higher liver fat content, independent of other metabolic risk factors. After adjustment for metabolic confounders, VAT not only retained a moderate positive correlation with hepatic fat content but also contributed approximately 50% of the explained variance, comparable to the share accounted for by the respective covariate, in both men and women. This finding aligns with the results reported by Chartrand et al. (*r* ≈ 0.55) [1], as well as with existing evidence identifying visceral adiposity as a central contributor to the pathogenesis and severity of MAFLD [16, 34]. This suggests an independent association between visceral fat volume and hepatic steatosis and supports its biological plausibility, as visceral fat drains directly into the portal circulation, delivering free fatty acids and pro-inflammatory adipokines to the liver [35]. Interestingly, the VAT-to-SFT ratio did not emerge as a significant predictor in our models for either sex. The limited variability of the ratio may reflect the parallel increase of visceral and subcutaneous fat in certain phenotypes — predominantly among individuals with overweight — which could attenuate the ratio’s discriminatory capacity. Nevertheless, the higher proportion of explained variance in women compared to men suggests a potentially greater relevance of this ratio in the female subgroup. Notably, other research has found that a higher VAT-to-SFT ratio is associated with MAFLD and correlates with the risk of advanced fibrosis, particularly among people without obesity [36]. Therefore, VAT/SFT ratio may be of limited utility in men in this cohort, as both visceral and subcutaneous fat contributed meaningfully to liver fat variance in this group. In women, by contrast, where subcutaneous fat played a smaller independent role, the ratio may have more discriminatory value—potentially reflecting a more risk-prone adipose distribution.

### Subcutaneous fat: A benign buffer or an additional risk?

#### Total fat depot

In contrast, the role of subcutaneous fat in hepatic steatosis is less well understood and some longitudinal studies suggest that greater subcutaneous fat may offset metabolic risk to a degree [37]. However, our findings add nuance and challenge the VAT-centric view by showing that total subcutaneous fat can influence liver fat. Total models including SFT showed similar total r values (>0.7) as VAT models, indicating that subcutaneous fat depots explained hepatic fat content almost as well as visceral fat. This significant predictive value of SFT suggests that individual differences in subcutaneous fat storage capacity may influence liver fat independently of visceral adiposity, with notable sex differences. In women, SFT exhibited the highest partial correlation with hepatic fat content among all fat depots (*r* = 0.51), yet accounted for only approximately one-fourth of the explained variance in hepatic fat according to the LMG analysis. By far the largest contributor in women was HC (73%), which diminished the effect of SFT. This suggests that in the female cohort, elevated cholesterol was a key determinant of liver fat content. Such finding aligns with the well-established link between dyslipidemia and NAFLD, although the relationship is likely bidirectional and should be interpreted accordingly [38]. Moreover, the stronger association between SFT and hepatic fat observed in women is likely correlational rather than causal.

In men, total SFT showed a weaker association with liver fat than in women, but explained a larger proportion of the variance (~ 42%). The remaining variance was largely attributable to diabetes. According to these results, subcutaneous fat does not appear irrelevant—particularly in men and especially in the presence of metabolic disturbances [39]. This aligns with large epidemiologic studies that noted that SAT area positively correlated with liver fat accumulation [40, 41]. Beals et al. also reported that SAT triglyceride synthesis is not impaired in NAFLD, whereas SAT fibrogenesis is markedly elevated and tightly linked to insulin resistance [9]. Conversely, a recent systematic review by Mátis et al. found that reductions in SAT were significantly associated with improvements in hepatic steatosis [42].

The sex-specific predominance of metabolic diseases observed in our study is biologically plausible. In a study by Arner et al., adipose tissue insulin resistance was more pronounced in men, attributed to less efficient insulin-mediated inhibition of adipocyte lipolysis, a higher basal lipolytic rate, and reduced insulin signaling in adipose tissue [43]. Notably, epidemiologic evidence remains heterogeneous, with some cohorts reporting stronger diabetes–NAFLD associations in women; thus, our observed sex differences should be interpreted cautiously, considering sample size, study design, and analytical approach [44]. While hypercholesterolemia is a recognized correlate of MASLD [38], it appeared more influential in women in our cohort, possibly reflecting postmenopausal alterations in cholesterol metabolism and estrogen-dependent regulation of hepatic lipid handling (e.g. estrogen reduces hepatic de-novo lipogenesis) [45, 46]. A study of Marchesini et al. also reported higher total cholesterol in women than in men [47].

#### Regional fat depots and skinfolds

Our sex-stratified analyses indicate that, in both sexes, the subcutaneous fat component most strongly associated with liver fat is located in the upper-body, whereas lower-body SFT contributes little unique variance. The study of Lee et al. hypothesized, that upper subcutaneous fat has a more systemic effect rather than locally [15]. However, associations and contributions were more strongly associated with liver fat in men than in women. Interestingly, Rodelo et al. reported that the odds of NAFLD were highest (OR ~ 18) in individuals with both metabolic syndrome (MetS) and abdominal SAT obesity, compared with MetS alone (OR ~ 10) or high SAT alone (OR ~ 5). This suggests a supra-additive interaction in which subcutaneous fat augments an already insulin-resistant state, thereby contributing to liver fat accumulation.

Additionally, the triceps skinfold was the only clearly useful single site in men, outperforming umbilical and far exceeding biceps and femoral. Importantly, this skinfold exhibited partial correlations, model R², and LMG contributions that were broadly comparable to VAT. This does not imply equivalence or causality, but within our sample it suggests that triceps thickness may function as a pragmatic indicator of liver-fat risk in men. However, thresholds and predictive utility will require confirmation in larger, prospective cohorts.

In women, the umbilical—and, to a lesser extent, the biceps—skinfolds were the only sites showing meaningful associations with liver fat, whereas the triceps was uninformative and the femoral site contributed minimally. A few studies have reported associations between subcutaneous fat thickness (e.g., suprailiac or triceps sites) and ultrasound-based estimates of hepatic steatosis [48–50]. These results align with our observations, supporting the relevance of subcutaneous fat distribution for hepatic lipid accumulation. However, to our knowledge, no prior work has systematically compared major fat depots and standardized skinfold sites in relation to MRI-derived hepatic fat content within a sex-stratified adult cohort.

These results suggest that total SFT largely mirrors the composite effect of its regional depots, integrating opposing associations from upper- and lower-body compartments. In summary, our results align with work emphasizing the primacy of visceral and trunk adiposity for hepatic steatosis, while lower-body subcutaneous fat contributed less variance to liver-fat accumulation [8, 51].

### Limitations

Although our cross-sectional design limits causal inference, we identified clear vulnerability patterns in this cohort. LMG contributions are model-, sample-, and prevalence-dependent and should be interpreted only within the context of this cohort. Future studies should validate these findings in larger samples and longitudinal designs to clarify causal pathways between fat distribution and hepatic lipid deposition to enhance generalizability. Extending analyses to disease states such as liver fibrosis and cirrhosis could yield further insights. The absence of significant association between self-reported physical activity and hepatic fat may reflect limitations in capturing intensity data and therefore exercise and metabolic capacity. FM was assessed using single-frequency rather than multi-frequency BIA, which may yield slightly different absolute values. Despite well-performing models, approximately 40–50% of liver fat variance remained unexplained, suggesting roles for unmeasured factors such as diet, genetics, lifestyle, or endocrine influences—warranting investigation in larger, sex-stratified MASLD studies.

## Conclusion

In summary, this study not only confirmed the pivotal role of visceral fat in both sexes but also highlighted its substantial relevance in early hepatic steatosis. For the first time, we provide sex-specific insights into the contribution of total and regional subcutaneous fat, including clinically applicable skinfold sites. We identified subcutaneous regions—particularly the upper-body and triceps in men—that exhibited associations comparable to the liver-fat–critical visceral compartment. To help bridge the gap between epidemiological research and clinical practice, future studies in larger cohorts should evaluate standardized and easily accessible skinfold measurements as low-cost tools for early risk screening in routine care.

## Supplementary Information

Supplementary Material 1.

## Data Availability

Data is provided within the manuscript. Additional datasets generated for this study are available from the corresponding author upon reasonable request. Methodological details are provided in the supplementary material.

## Abbreviations

AASLD: American association for the study of liver diseases
aHT: Arterial hypertension
BIA: Bioelectrical impedance analysis
BMI: Body mass index
BSA: Body surface area
DM: Diabetes mellitus
EASL: European association for the study of the liver
FM: Fat mass
HC: Hypercholesterolemia
ICC: Intraclass correlation coefficient
ISAK: International Society for the advancement of kinanthropometry
LF: Liver fat
LMG: Lindeman–Merenda–Gold (method for variance decomposition)
MASLD: Metabolic dysfunction–associated steatotic liver disease
MRI: Magnetic resonance imaging
NAFLD: Non-alcoholic fatty liver disease
SAT: Subcutaneous adipose tissue (abdominal)
SFT: Subcutaneous fat tissue (total and regional subcutaneous fat)
SFT_arms: Subcutaneous fat tissue of arms
SFT_biceps: Subcutaneous fat depth at biceps skinfold site
SFT_femoral: Subcutaneous fat depth at femoral/thigh skinfold site
SFT_lowerBody: Subcutaneous fat tissue of legs and gluteal sides
SFT_upperBody: Subcutaneous fat tissue of trunk
SFT_triceps: Subcutaneous fat depth at triceps skinfold site
SFT_umbilical: Subcutaneous fat depth at abdominal/umbilical skinfold site
VAT: Visceral adipose tissue
VAT/SFT: Visceral-to-subcutaneous fat ratio
