# Supplementary material for: The role of adipose tissue in liver fat accumulation: a sex-specific analysis in an exploratory cross-sectional study
Source: Lipids Health Dis. 2025 Dec 1;24:376. doi: 10.1186/s12944-025-02809-x (PMC12670730; doi:10.1186/s12944-025-02809-x)
Supplement: Supplementary file 1 — Supplementary Material 1. [file 12944_2025_2809_MOESM1_ESM.docx]

**Supplementary Material**

Liver Fat Assessment

Fat–water separation methods such as Dixon rely on chemical shift imaging (CSI), exploiting the frequency difference (~3.5 ppm) between water and fat [1]. At 1.5T, this shift equals ~217Hz for the main fat signal [2]. As a result, water and fat signals align in-phase (IP) at multiples (k) of ~ 4.6ms, and out-of-phase (OP) at (~2.3 + k * 4.6ms) [1].

Gradient echo sequences like Dixon or bFFE are well-suited for exploiting this frequency offset, as they allow the acquisition of both in-phase and out-of-phase images [3]. However, the bFFE protocol (flip angle = 20° [4], TR = 120 ms, 10 echo times with echo spacing of 0.84ms starting at TE=1.19ms) introduces confounding factors requiring targeted post-processing. The available steady-state magnetization per echo train depends on the T1 relaxation time of the tissue. Given echo times much shorter than effective T1, its influence on decay was neglected. Corrections were applied to account for fat signal intensity discrepancies and artifacts arising from the sequence’s bipolar readouts. The high number of echo points allowed for more precise modeling of relaxation decay and fat spectrum interferences [5].

Short TEs require bipolar readout gradients [6], but residual B0 and gradient-related artifacts (e.g., non-linearity) caused alternating deviations between even and odd echoes, also seen in echo-planar imaging (EPI) sequences [5, 7]. To reduce sensitivity to magnetic field inhomogeneity, shimming was performed before acquisition, and short TRs were applied. To minimize susceptibility artifacts, local ROI analysis was used. ROIs (~1 cm²) were placed in liver segments 6/7, avoiding large vessels, as this region correlates well with liver biopsy results [8]. Placement was performed by a radiologist with over 30 years of experience. The high intra- and inter-rater reliability of this method has been demonstrated in previous studies [9].

Mean signal values were extracted across all echoes per ROI and normalized by the maximum signal value across the echoes, since absolute signal values depend on setup- and scanner-specific factors and carry no quantitative meaning. In addition to the inherent alternating signal phase caused by the readout polarity change (see Figure S1), the signal amplitude depends on the spatial position and echo number [10]. Even and odd echoes were modeled separately; cubic spline interpolation generated continuous curves respectively, which were averaged to reconstruct the unbiased signal, assuming a linear phase relationship [6]. The resulting midline reflects the corrected signal. The processing steps are illustrated in Figure 1. One male participant was excluded from the analysis due to localized fat inhomogeneity.

According to Schwenzer et al., the average decay in liver is 33.87 [s^-1^] for women and 39.37 [s^-1^] for men [11]. Consequently, we introduced the effective relaxation into the modeling ($R_{2}^{*}$). To extract the fat fraction, we included six dominant fat peaks from the fat spectrum identified for liver tissue in Hamilton et al. with f_k_ = [242.7, 217.1, 166.1, 124.5, 31.9, -38.3] Hz, and respective amplitudes αk = [0.087, 0.693, 0.128, 0.004, 0.039, 0.048], ensuring $\sum_{k=1}^{6} a_{k} = 1$ [12, 13]

Introducing the fat fraction (f*)*, we define the signal contributions of fat $S_{f}=fS_{0}$ and water $S_{w}=\left( 1-f \right)S_{0}$, where $S_{0}$ represents the total signal. This allows us to describe the signal time evolution using the following model:

|  | $S\left( t \right)=S_{0}e^{-R_{2}^{*}t}\left( 1-f+f\sum_{k=1}^{6} \left\vert\alpha_{k}e^{\left( i\omega_{k}t \right)} \right\vert\right)$ |  |  |
| --- | --- | --- | --- |

The model we used after correction is visualized in Figure S1. The fat fraction was obtained by performing a least-squares fit of the model to the data points from each ROI and participant. The regression was carried out using polars and sciPy within Python (Version 3.10.0, https://www.python.org).


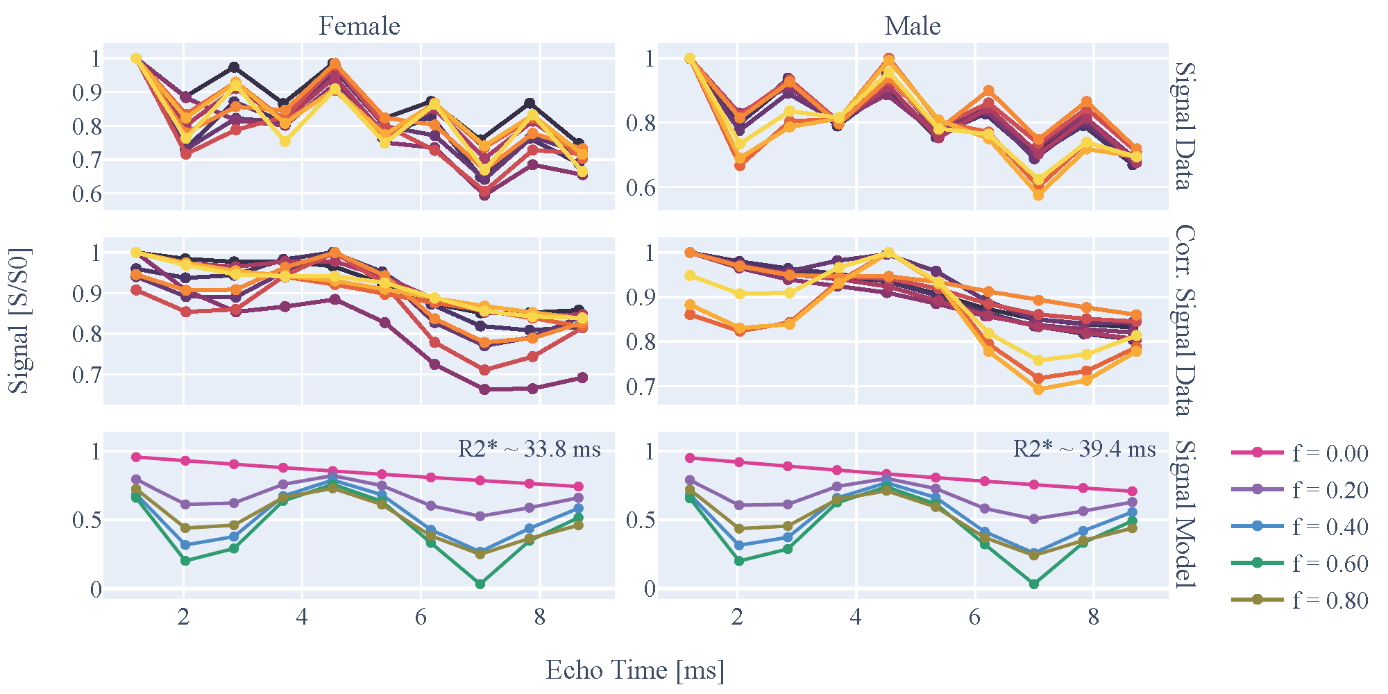


**Figure S1 bFFE signal and signal model for liver.** bFFE Signal for liver (top row) for 10 randomly selected female (left) and male (right) participants. The steady state phase modulation between the echoes creates a characteristic alternating signal pattern. Any volume-specific offset can change the alternation of this pattern, making it dependent on the ROI position. The signal decay towards later echoes is visible. If the fat contribution to liver tissue signal is considerable, the fat spectrum modulation can be observed in the signal creating a sinusoidal shape. This characteristic pattern is more prominent after correction (middle row). The model used for estimating fat and decay parameters can be seen in the bottom row using S0 = 1 and R2* ~ 33.8 [s^-1] (left) and R2* ~ 39.4 [s^-1] (right) in Equation (1) in supplemental material. The R2* chosen is roughly the expected decay for liver for female and male participants taken from Schwenzer et al. [11]. Corr: corrected

References

1. Reeder SB, Cruite I, Hamilton G, Sirlin CB. Quantitative assessment of liver fat with magnetic resonance imaging and spectroscopy. J. Magn. Reson. Imaging. 2011;34:729–49. doi:10.1002/jmri.22580.

2. Xiang Q-S. Two-point water-fat imaging with partially-opposed-phase (POP) acquisition: an asymmetric Dixon method. Magn Reson Med. 2006;56:572–84. doi:10.1002/mrm.20984.

3. Bray TJ, Chouhan MD, Punwani S, Bainbridge A, Hall-Craggs MA. Fat fraction mapping using magnetic resonance imaging: insight into pathophysiology. Br J Radiol. 2018;91:20170344. doi:10.1259/bjr.20170344.

4. Martin DR, Danrad R, Herrmann K, Semelka RC, Hussain SM. Magnetic resonance imaging of the gastrointestinal tract. Top Magn Reson Imaging. 2005;16:77–98. doi:10.1097/01.rmr.0000179461.55234.7d.

5. Henze Bancroft LC, Strigel RM, Hernando D, Johnson KM, Kelcz F, Kijowski R, Block WF. Utilization of a balanced steady state free precession signal model for improved fat/water decomposition. Magn Reson Med. 2016;75:1269–77. doi:10.1002/mrm.25728.

6. Lu W, Yu H, Shimakawa A, Alley M, Reeder SB, Hargreaves BA. Water-fat separation with bipolar multiecho sequences. Magn Reson Med. 2008;60:198–209. doi:10.1002/mrm.21583.

7. Dierkes T, Neeb H, Shah N. Distortion correction in echo-planar imaging and quantitative T2* mapping. International Congress Series. 2004;1265:181–5. doi:10.1016/j.ics.2004.03.041.

8. Bhat V, Velandai S, Belliappa V, Illayraja J, Halli KG, Gopalakrishnan G. Quantification of Liver Fat with mDIXON Magnetic Resonance Imaging, Comparison with the Computed Tomography and the Biopsy. J Clin Diagn Res. 2017;11:TC06-TC10. doi:10.7860/JCDR/2017/26317.10234.

9. Leitão H, Paulino C, Rodrigues D, Gonçalves S, Marques C, Carvalheiro M, et al. MR Fat Fraction Mapping: A Simple Biomarker for Liver Steatosis Quantification in Nonalcoholic Fatty Liver Disease Patients. Academic radiology. 2013;20:957–61. doi:10.1016/j.acra.2013.05.004.

10. Scheffler K. Fast frequency mapping with balanced SSFP: theory and application to proton-resonance frequency shift thermometry. Magn Reson Med. 2004;51:1205–11. doi:10.1002/mrm.20081.

11. Schwenzer NF, Machann J, Haap MM, Martirosian P, Schraml C, Liebig G, et al. T2* relaxometry in liver, pancreas, and spleen in a healthy cohort of one hundred twenty-nine subjects-correlation with age, gender, and serum ferritin. Invest Radiol. 2008;43:854–60. doi:10.1097/RLI.0b013e3181862413.

12. Yu H, Shimakawa A, McKenzie CA, Brodsky E, Brittain JH, Reeder SB. Multiecho water-fat separation and simultaneous R2* estimation with multifrequency fat spectrum modeling. Magn Reson Med. 2008;60:1122–34. doi:10.1002/mrm.21737.

13. Hamilton G, Yokoo T, Bydder M, Cruite I, Schroeder ME, Sirlin CB, Middleton MS. In vivo characterization of the liver fat ¹H MR spectrum. NMR Biomed. 2011;24:784–90. doi:10.1002/nbm.1622.

| Table S1 Sex-specific Pearson correlations and contributions (LMG) of adipose tissue depots and all metabolic factors to liver fat | | | | | | | |  |  |
| --- | --- | --- | --- | --- | --- | --- | --- | --- | --- |
| **Model** | **Total** | **Partial** | **Total** | **Portion of Total R²** | | | | | |
|  | **r** | **r** | **R²** | **Fat depot** | **DM** | **HC** | **aHT** | **PA** | **Age** |
| **male (n=22)** |  |  |  |  |  |  |  |  |  |
| VAT/BSA + DM + HC + aHT + PA + Age | 0.78* | 0.56* | 0.61 | 0.26 | 0.18 | 0.03 | 0.06 | 0.01 | 0.08 |
| SFT/BSA + DM + HC + aHT + PA + Age | 0.71* | 0.34 | 0.50 | 0.14 | 0.24 | 0.03 | 0.04 | 0.01 | 0.04 |
| FM/BSA + DM + HC + aHT + PA + Age | 0.70* | 0.29 | 0.48 | 0.18 | 0.18 | 0.03 | 0.04 | 0.01 | 0.04 |
| **female (n=24)** |  |  |  |  |  |  |  |  |  |
| VAT/BSA + DM + HC + aHT + PA + Age | 0.79* | 0.24 | 0.62 | 0.16 | 0.07 | 0.21 | 0.03 | 0.07 | 0.07 |
| SFT/BSA + DM + HC + aHT + PA + Age | 0.83* | 0.46* | 0.68 | 0.08 | 0.08 | 0.32 | 0.04 | 0.07 | 0.08 |
| FM/BSA + DM + HC + aHT + PA + Age | 0.81* | 0.41 | 0.66 | 0.11 | 0.08 | 0.29 | 0.04 | 0.07 | 0.07 |

VAT: visceral adipose tissue; SFT: total subcutaneous fat; FM: whole-body fat mass; DM: diabetes mellitus; HC: hypercholesterolemia; PA: physical activity; aHT: arterial hypertension; Total r: correlation of the combination of depot plus confounder; Total R²: LF variance explained by the combination of depot plus confounder; partial r: confounder- adjusted correlation; portion of Total R²: LF variance explained by this specific variable calculated via LMG method; * significant values, α<0.05, one-sided
